# Supplementary material for: Consistent Biomarkers and Related Pathogenesis Underlying Asthma Revealed by Systems Biology Approach
Source: Int J Mol Sci. 2019 Aug 19;20(16):4037. doi: 10.3390/ijms20164037 (PMC6720652; doi:10.3390/ijms20164037)
Supplement: Supplementary file 1 [file ijms-20-04037-s001.zip › ijms-566768-supplementary-XML/Supplementary Material S2-Reports of Quality control for ten datasets.zip!/Supplementary Material S2-Reports of Quality control for ten datasets/QC_report_for_GSE104468/ma.pdf]

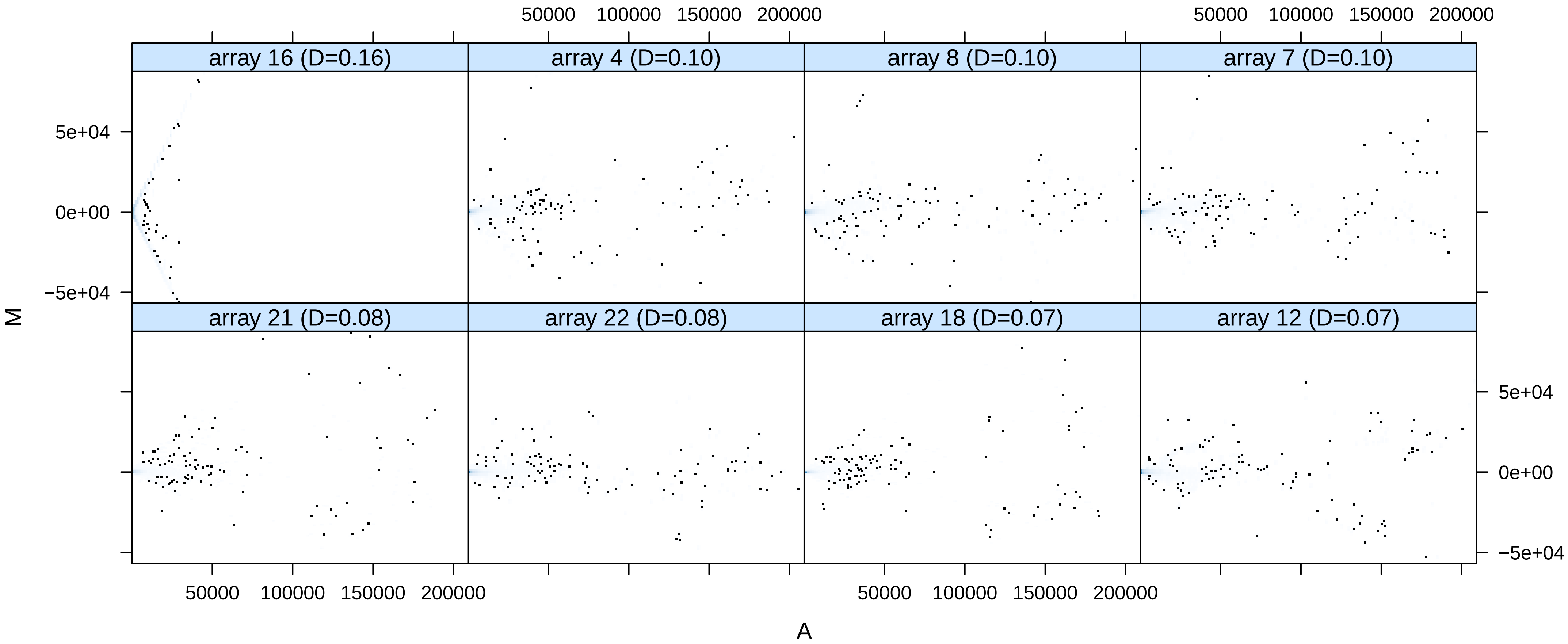
array 16 (D=0.16)
array 4 (D=0.10)
array 8 (D=0.10)
array 7 (D=0.10)
array 21 (D=0.08)
array 22 (D=0.08)
array 18 (D=0.07)
array 12 (D=0.07)
M
A
5e+04
0e+00
−5e+04
50000
100000
150000
200000
